# Chinese medicine and biomodulation in cancer patients—Part one

**DOI:** 10.3747/co.2008.197

**Published:** 2008-01

**Authors:** S.M. Sagar, R.K. Wong

**Affiliations:** * Departments of Oncology and Medicine, McMaster University; Juravinski Cancer Program, Hamilton Health Sciences Corporation; and The Brain–Body Institute, St. Joseph’s Healthcare System, Hamilton, ON

**Keywords:** Chinese medicine, herbs, acupuncture, supportive care, immunity, research

## Abstract

Traditional Chinese Medicine (tcm) may be integrated with conventional Western medicine to enhance the care of patients with cancer. Although tcm is normally implemented as a whole system, recent reductionist research suggests mechanisms for the effects of acupuncture, herbs, and nutrition within the scientific model of biomedicine. The health model of Chinese medicine accommodates physical and pharmacologic interventions within the framework of a body–mind network. A Cartesian split does not occur within this model, but to allow for scientific exploration within the restrictions of positivism, reductionism, and controls for confounding factors, the components must necessarily be separated. Still, whole-systems research is important to evaluate effectiveness when applying the full model in clinical practice. Scientific analysis provides a mechanistic understanding of the processes that will improve the design of clinical studies and enhance safety. Enough preliminary evidence is available to encourage quality clinical trials to evaluate the efficacy of integrating tcm into Western cancer care.

## 1. INTRODUCTION

Recent evidence suggests that many therapies from Traditional Chinese Medicine (tcm) can be effective for the supportive care of cancer patients. The present narrative review of the published literature provides various levels of evidence that support further research into a developing model of integrative care.

A plethora of well-designed preclinical studies are available, but randomized controlled trials are scarce. This situation is not a consequence of inadequate mechanistic knowledge; rather, it is secondary to challenges in clinical trial design and funding. Most published clinical studies are at evidence level iii—in other words, they are trials without randomization or they involve single group pre–post, cohort, time series, or matched case–control studies. We emphasize level i and ii evidence from well-designed randomized controlled trials of appropriate size. In view of the paucity of quality data providing level i and ii evidence, meta-analysis is usually not possible. In view of the promising mechanistic knowledge and clinical observations, we encourage welldesigned randomized controlled trials.

## 2. THE CONTRAST BETWEEN TCM AND BIOMEDICINE

It is important that tcm as a whole system not be discounted simply because an individual study that evaluates a single intervention for a specific disease entity is negative. The same mindset obviously applies to Western biomedical medicine. A negative drug study does not negate the whole of biomedicine. Moreover, the situation is complicated by the different definitions of disease in Eastern and Western medicine, and the philosophy of concurrent multiple interventions, individualization, and modification of approach during a course of treatment.

The challenge for tcm is to develop repeatable and provable outcomes, standardization, and quality assurance. The scientific bases of herbs and acupuncture are rapidly being established, but well-designed, pragmatic, controlled clinical studies are lacking in most domains. To enhance patient care, tcm could be practiced alongside conventional Western medicine. The philosophy of tcm proposes novel hypotheses that will support the development of a science-based holistic medicine.

Biomodulation is the reactive or associative adjustment of the biochemical or cellular status of an organism. Most modulation events describe an interaction in which a molecule (modulating entity) alters the ability of an enzyme to catalyze a specific reaction. In the context of cancer, biomodulation includes the use of a substance to augment the host’s antitumour response, including immunotherapy. It encompasses the regulation of innate electrophysiologic, chemical, and molecular pathways through relatively low-intensity physical and chemical interventions. In contrast to conventional biomedicine (drugs, for example), therapies such as herbs or their extracts are a mixture of chemicals administered at relatively low doses over a prolonged period of time. Acupuncture results in low-level electrochemical changes in the soft tissue fascia.

In tcm, the practice model includes the targeting of individual imbalances using a diagnostic philosophy derived from cumulative clinical observation. In Western medicine, the combination of tcm herbs with drug therapies is controversial, because knowledge of whether the drug is favourably enhanced or whether adverse effects occur is lacking. Future clinical research needs to evaluate the combinations using initial data from the preclinical studies, some of which report favourable synergy. Parts one and two of this article will both describe examples of biomodulation and the results of combining tcm with biomedicine. Part one discusses the broad principles of tcm; part two discusses its potential clinical applications in oncology.

## 3. DISCUSSION

### 3.1 Cancer As a Systemic Disease

In Western medicine, cancer is conventionally considered from the somatic viewpoint: a clone of cells that have outgrown environmental constraints and control mechanisms. The cells are abnormal and are considered to be foreign to the body.

The main philosophy of cancer treatment is direct annihilation of the cancer cells, using aggressive and destructive therapies. Traditional Chinese Medicine emphasizes the importance of the body–mind communication network. By demonstrating the effects of emotions on cellular immunity and other mechanisms, the science of psychoneuroimmunology has set out a potential physiologic basis for cancer cell progression.

In tcm, the development of cancer is viewed as one of the presenting features of a syndrome representing an imbalance of the whole body–mind network 1. In other words, cancer is a systemic disease from the start, and the terrain is considered to be as important as the tumour itself 2. Practitioners of tcm believe that if the body–mind network is strengthened and rebalanced, the normal pattern will be restored, thereby helping to resolve the cancer. This concept is currently being incorporated into a more holistic science, in which the whole picture is as important as the parts. To quote Hanahan and Weinberg 3, “The metaphors used to conceptualize cancer cell function will also shift dramatically. For decades now, we have been able to predict with precision the behavior of an electronic integrated circuit in terms of its constituent parts—its interconnecting components, processing, and emitting signals. Having fully charted the wiring of every cellular signaling pathway, it will be possible to lay out the complete ‘integrated’ circuit. We will then be able to apply the tools of mathematical modelling to cancer cells. With holistic clarity of mechanism, cancer prognosis and treatment will become a rational science.”

Recent evidence suggests that bone marrow stem cells may play a significant role in the perpetuation of some cancers, including the production of proangiogenic peptides. Thus Western science is now exploring the possibility that both hematologic and solid cancers may sometimes be a systemic disease from the outset 4–6.

### 3.2 The Body–Mind Network

Traditional Chinese Medicine recognizes that the human being functions as a body–mind network 7. The philosophy of tcm analyzes the “process” of body–mind communication, rather than a “snapshot” of structural material entities such as molecules. If Western medicine is viewed as the hardware of a computer, then tcm could represent the software—although humans are more complex than the computer metaphor suggests, because the “software and hardware” can modify each other 8. Moreover, tcm theory recognizes a correspondence between patterns of information that are independent of the carrier of the information. For example, a *pattern* of information may be similar regardless of whether it is mediated by pulses of hormones and neuropeptides or by an electrophysiologic frequency pattern 9–13.

Acupuncture stimulation of specific points on the body induces not only local release of cytokines, but also electrophysiologic communication through the fascia and peripheral nerves. Propagation of the electrophysiologic waves eventually releases neuropeptides (such as somatostatin and vasoactive intestinal peptide) within the central nervous system 14,15. The totality of communication is thus multifactorial and time-dependent. The effects of short-term stimulation may be quite different from those of a longer course of acupuncture therapy that could, perhaps, induce neural remodelling 16.

The body–mind information system may be partly regulated by the relative contributions of the sympathetic and parasympathetic components of the autonomic nervous system. This model of regulation corresponds to the tcm concept of balance between yin and yang, which represents a pattern of information, rather than concrete material entities. An analysis of the pulse by the classical Chinese technique may indicate the relative imbalance, as has been demonstrated indirectly by using appropriate computer software to produce a spectral analysis of electrocardiograms. Acupuncture has been shown to rebalance the relative contributions of the sympathetic and parasympathetic nervous systems 17. Patterns of information transfer may interact to entrain and reinforce information flow in a complex dynamical system 18–20. The system is an autopoietic process. In other words, it can recreate itself and evolve through learning, so that the body can adapt to changing circumstances.

When an individual is healthy, communication flows freely between systems in a complex, nonlinear, heterarchical and hierarchical process of information transfer through physiologic interactions. Metaphorically, mind–body communication is represented by an informatics system of energy in motion—in other words, “e-motion.”

Cancer may be associated with a disturbance in the information flow, manifested by an overplastic system that loses process structure and becomes irreversibly chaotic 21 in association with local electrophysiologic changes 22. Experiments in rats show that chronic restraint stress promotes lymphocyte apoptosis through modulation of *fas* (sometimes called *CD95*) gene expression via a pathway that involves opioid receptors 23. In other words, stress can influence both the function and the structure of the nervous system, in turn possibly modulating lymphocyte gene expression, thereby influencing immunity and resistance to cancer. Intervention with a technique such as acupuncture may restore the general imbalance in information flow—for example, by rebalancing the sympathetic and parasympathetic components of the autonomic nervous system 24,25. The same model of interaction with subconscious stress systems helps to achieve an understanding of how the compassionate intentionality of a healer can restore health through entrainment and normalization of an imbalanced system 26.

Although these communication pathways may play a very limited role for advanced cancer patients, calming the stress system could conceivably slow cancer progression and recurrence by reducing the numbers of immunocytes, such as myeloid suppressor cells and T regulator cells, that protect cancer cells 27. Understanding these processes requires consideration of systems outside the current reductionist pharmacologic model. The multiple and widely distributed systems involved in psychoneuroimmunology are complex and require holistic systems research. Development of computerized algorithmic modelling of dynamical systems and networks will be essential to reaching an understanding of the concurrent, synergistic contributions of multiple systems on body–mind outcomes 28–32.

The beauty of the body–mind network model is that it can combine constitutional personality factors (such as emotions and feelings) with bodily symptoms into a single diagnostic and treatment paradigm. In tcm terms, this diagnostic model is represented by patterns of disharmonies in the main organ systems and by abnormalities of *qi* (energy flow), essence (energy reserves), blood, heat, and moisture. Interestingly, the association within the tcm model of cancer predisposition with rising *qi* (*sheng qi*) or “liver fire” (representing anger) shows some correspondence with the controversial scientific evidence that repressed anger suppresses the immune system and may increase the risk of breast cancer in the so-called type C personality 33–35.

### 3.3 Biologic Effects of Acupuncture

We propose that the clinical intervention of acupuncture allows practitioners to modulate communication within the body–mind network through concurrent changes in multiple signalling pathways. Most evidence suggests that acupuncture modulates neurotransmitters, cytokines, and neuropeptides through electrophysiologic changes in the nervous system 36,37. As the activity of afferent peripheral nerves is either stimulated or suppressed, interactions with the brain stem, hypothalamus, limbic system, and autonomic nervous system occur 38–47. Acupuncture has also been reported to modify the somatic electromagnetic field 48, but research in that area remains quite controversial.

Recent research suggests that the initial transduction of the acupuncture needle is through myofascial tissue planes. The network of acupuncture points and meridians can be viewed as a representation of the network formed by interstitial connective tissue. Langevin and Yandow mapped acupuncture points in serial gross anatomic sections through the human arm 49. In post-mortem tissue sections, they found an 80% correspondence between the sites of acupuncture points and the location of intermuscular or intramuscular connective tissue planes. The surface points appear to connect to a web of vessels that cover internal organs, as demonstrated by special dna staining 50. The *de qi* (“needle grasp”) effect may be attributable to mechanical coupling between the needle and the connective tissue with winding of tissue around the needle during needle rotation. Needle manipulation could transmit a mechanical signal to connective tissue cells via mechanotransduction 51. That signal may be converted into an electrophysiologic response through a change in electrical impedance that spreads through the connective tissue planes, interacts with cellular genomic expression, and releases local cytokines and other messenger molecules that may initiate neurologic transmission 52.

In view of the power of the placebo effect, a sham acupuncture control arm is required as a standard for randomized controlled trials of acupuncture. Sham needles have been validated to a limited extent. The results may depend on whether the subject has had past experience of acupuncture. These devices withdraw a blunt needle back into the sheath during application, a sham technique that is more valid than using superficial penetration of the skin or randomly assigning points away from known meridians.

Evaluation of the widely used Streitberger sham needle concluded that most patients were unable to discriminate between the needles by actual penetration; however, nearly 40% perceived a difference in treatment type between sham and actual needles 53. The fact that nearly 40% of subjects perceived dissimilarities in the two interventions raises some concerns with regard to the wholesale adoption of this instrument as a standard acupuncture placebo. The authors of the study concluded that further work on between-tester reliability and standardization of technique is highly recommended before the Streitberger needle can be confidently used in future studies.

### 3.4 Pharmacology of Chinese Herbs

In tcm, herbs are used in combinations meant to enhance their benefits and simultaneously to reduce their side effects 54. In effect, multiple low-dose pharmacologic agents are being administered synergistically. Western medicine usually focuses on maximally tolerated doses of single agents, but chemotherapy drugs are typically combined, usually to minimize drug resistance. The consequence of such combination can be a further increase in drug toxicity. According to tcm practitioners, combinations of herbs can reduce the side effects of anticancer drugs; however, further research with herbs is indicated to determine their pharmacokinetic interactions with pharmaceutical drugs and their potential adverse effects.

**FIGURE 1:**
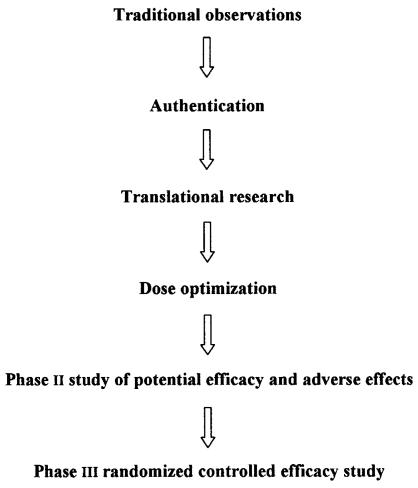
Systematic scientific approach for introducing Chinese herbs into Western clinical practice.

A scientific approach to introducing Chinese herbs into Western practice involves a rigorous and systematic approach to phytochemical profiling, quality control, preclinical evaluation, safety evaluation, and phase i–iii clinical trials (Figure 1). More data are required to establish optimal combinations of herbs that produce synergistic activity. Traditionally, Chinese herbs have been used as complex mixtures.

One study evaluated the dna microarray data for 12,600 genes to examine the antiproliferative activity of the single herb *Coptidis rhizoma* and 8 constituent molecules against 8 human pancreatic cancer cell lines 55. It identified 27 genes showing a strong correlation with the 50% inhibitory dose (ID^50^) of *C. rhizoma* after a 72-hour exposure. Hierarchical cluster analysis used correlation coefficients between expression levels of those 27 genes and the ID^50^ of each constituent molecule to classify the test molecules into two clusters: one consisting of *C. rhizoma* and berberine, and the other consisting of the remaining seven molecules. The results suggest that one specific phytochemical, berberine, can account for most of the antiproliferative activity of *C. rhizoma* and that dna microarray analyses can be used to improve our understanding of the actions of an intact herb.

In contrast, there can be merit in using combinations of herbs and their derivatives. For example, PHY106 (*Radix scutellariae*, *Paeonia lactiflora* Pall, *Fructus ziziphi, Radix glycyrrhizae*) is an authenticated combination of herbs that may increase the efficacy and reduce the adverse effects of the cytotoxic drug capecitabine, which is used in treating colorectal cancer 56. The biotechnology company PhytoCeutica (New Haven, CT, U.S.A.) has systematically profiled the phytochemical content of each herb and tested various combinations at the preclinical stage to optimize the complex mixture. Quality has been assured by establishing profiles using chemical chromatography and spectroscopy, together with biologic, proteomic, and genomic profiling. Through these techniques, Phytoceutica developed a Phytomics Similarity Index. Interestingly, their modern technological approach confirms the tcm theory of optimizing efficacy through a hierarchy of herb combinations. The PHY106 combination enhances antitumour activity, reduces toxicity, and enhances the pharmacokinetics of the chief herb (the “Emperor herb”). Mechanisms include inhibiting drug resistance proteins that may decrease absorption, inhibiting cytochrome P450 enzymes that metabolize phytochemicals, inhibiting microfloral β-glucuronidase, chemical stabilization, and modification of solubility.

Initial clinical trials demonstrate a reduction of gastrointestinal toxicity and enhancement of the tumoricidal effect of the chemotherapy. Many preclinical studies are now demonstrating that specific combinations of Chinese herbs can be synergistic with cytotoxic chemotherapy through both pharmacodynamic and pharmacokinetic interactions. For example, *Phellinus linteus* is a mushroom that consists mainly of polysaccharides. It sensitized an *in vitro* prostate cancer cell line to apoptosis induced by doxorubicin 57. At relatively low doses, neither *P. linteus* nor doxorubicin independently induced apoptosis in the cells. However, combination treatment with low doses of both agents resulted in a synergistic effect on the induction of apoptosis. Together with doxorubicin, *P. linteus* has a synergistic effect to activate caspases in lncap prostate cancer cells. Sensitization can be obtained at subtoxic concentrations of doxorubicin. As an apoptotic synergizer for conventional chemotherapeutics such as doxorubicin, *P. linteus* can reduce normal-tissue toxicity and increase therapeutic gain.

Gene expression profiling coupled with promoter assays can evaluate the effect of a herbal mixture on cancer. Such an approach may be used for the standardization of herbal extract activity. An example is the comparison of the gene profile of PC-SPES with that of PC-CARE, a product with a similar herbal composition. Early studies showed that PC-SPES contains estrogenic organic compounds, and such compounds are known to have an impact on prostate cancer. An important question is whether those compounds are the primary drivers of the gene profile. The data indicate that the gene expression profiles of lncap human prostate cancer cells in response to PC-SPES are different from those produced when diethylstilbestrol (des), a synthetic estrogen, is used, suggesting that the estrogenic moieties within PC-SPES do not drive the relevant expression signature 58. In contrast, the expression profile of the cells in the presence of PC-CARE is almost identical to that observed with des, indicating that mixtures containing similar herbs do not necessarily result in similar biologic activity.

To validate the expression profiling data, the investigators evaluated the protein expression and promoter activity of prostate-specific antigen (psa), a gene induced by PC-SPES, but repressed by des. To gain a mechanistic understanding of different effects of PC-SPES and des on psa expression, lncap cells were transiently transfected with wild-type and mutagenized psa promoter, androgen response element (are) concatemers, and appropriate controls. The evidence suggested that ares ii and iii within the promoter region are responsible for the suppressive effects of des and stimulatory effects of PC-SPES. In the case of des, the effects on psa transcription are specific to are, but PC-SPES affects the promoter non-specifically. The expression profiling, coupled with mechanistic target validation, yields valuable clues as to the mode of action of complex botanical mixtures and provides a new way to objectively compare mixtures that contain similar components either for effect or for quality assurance before they are used in clinical trials. In the case of PC-SPES, the effectiveness of the complex mixture of herbs has been shown in a randomized controlled clinical trial to be more effective than des alone 59.

Current technology is demonstrating the multidimensional activities of Chinese herbs as anticancer agents. Many are potent antioxidants that can induce apoptosis; others are anti-angiogenic agents 60,61. Many of the herbs that are traditionally considered effective against cancer cells are now being shown to have subtle effects on genetic expression; they may play a key role in anticancer treatment through synergistic activity with cytotoxic agents and maintenance regimens for prevention of recurrence 62.

Future research should maximize the use of technology to validate scientific mechanisms for quality assurance, safety, optimization, and clinical effectiveness within the modern cancer treatment environment 63. Rigorous adherence to the development of research protocols and standards of reporting are necessary, as stated by a recent publication of the Consolidated Standards of Reporting Trials group, which establishes the standards for clinical trials of natural health products 64. Further development of Chinese herbal medicine for administration in Western cancer clinics will require both international collaboration and an improved working relationship between national governments, industries, and universities.

## 4. CONCLUSIONS

The health model of Chinese medicine accommodates physical and pharmacologic interventions within the framework of a body–mind network. A Cartesian split does not occur within this model, but to allow for scientific exploration within the restrictions of positivism, reductionism, and controls for confounding factors, separation of the components is necessary. To evaluate effectiveness when applying the full model in clinical practice, whole-systems research is important. Considering relevant individual processes and contextual factors enhances the external validity of clinical research 65. A whole system is not like a drug with a specific action; rather, it produces effects that involve global shifts across multiple subsystems of the individual 66. However, at the opposite end of the epistemologic spectrum, scientific dissection enables a mechanistic understanding of the processes that will improve the design of clinical studies and enhance safety.

Part two of our review will dissect out some of the specific roles for tcm in biomodulation from a mechanistic Western science perspective.
